# HLA‐A*02:1227 and HLA‐A*36:16N, Two Novel HLA‐A Alleles Identified in Brazilian Bone Marrow Donors

**DOI:** 10.1111/tan.70690

**Published:** 2026-03-25

**Authors:** Augusto Cesar Soares dos Santos Júnior, Cíntia Keila Fabreti de Oliveira, Raquel Aparecida Fabreti‐Oliveira

**Affiliations:** ^1^ Department of Nephrology Hospital das Clínicas EBSERH, Federal University of Minas Gerais Belo Horizonte Minas Gerais Brazil; ^2^ Postgraduate Program in Health Sciences, Faculty of Medical Sciences of Minas Gerais Belo Horizonte Minas Gerais Brazil; ^3^ Department of Molecular Biology IMUNOLAB, Laboratory of Histocompatibility Belo Horizonte Minas Gerais Brazil

**Keywords:** bone marrow donor, HLA polymorphism, HLA‐A, NGS, REDOME

## Abstract

Identification of the novel HLA‐A*02:1227 and HLA‐A*36:16N alleles in Brazilian bone marrow donors.

The HLA genes are highly polymorphic and play a central role in allograft rejection and graft survival [1]. To date, more than 29,854 classical HLA class I alleles have been described, including 9022 HLA‐A alleles according to version 3.63.1 (February 2026) of the IPD‐IMGT/HLA Database [2]. Among these, 481 HLA‐A null alleles have been identified. This extensive polymorphism arises from several genetic mechanisms, including single nucleotide polymorphisms (SNPs), insertions and deletions and gene conversion events [3]. In some cases, SNPs introduce premature stop codons, resulting in null HLA alleles. Most null alleles fall outside the common, intermediate or well‐documented (CIWD) categories [4]. Failure to identify null HLA alleles may lead to inaccurate HLA matching, with potential clinical implications in transplantation. In this report, we describe two novel HLA alleles, now named HLA‐A*02:1227 and HLA‐A*36:16N, identified by next‐generation sequencing (NGS) in two unrelated volunteer donors from the Brazilian Registry of Volunteer Bone Marrow Donors (REDOME). Written informed consent was obtained from both donors in accordance with institutional and national ethical guidelines.

Genomic DNA was extracted from peripheral blood samples, and initial HLA typing was performed using NGS with the AllType NGS 11 Loci kit (One Lambda, Canoga Park, CA, USA), following the manufacturer's instructions and the methodology validated by Fabreti‐Oliveira et al. [5]. Sequencing was carried out on the Ion Torrent S5 platform (Thermo Fisher Scientific, Waltham, MA, USA) and the DNBSEQ‐G99 platform (MGI, Shenzhen, China). Data were analysed using TypeStream Visual software version 3.1.0 (One Lambda). To confirm the novel sequences and exclude technical artefacts, sequence‐based typing (SBT) was subsequently performed using the SeCore HLA SBT kit (One Lambda) on an ABI 3730 Genetic Analyzer (Applied Biosystems, Thermo Fisher Scientific). Sequence analysis using uType software version 7.3 confirmed the novel allelic assignments.

The allele HLA‐A*02:1227 was identified in an individual with the following HLA typing: HLA‐A*02:1227, 11:01:01; ‐B*15:01:01, 53:01:01; ‐C*01:02:01, 04:01:01; ‐DRB1*01:02:01, 04:03:01; DRB4*01:EUHVF; ‐DQA1*01:01:02, 03:01:01; ‐DQB1*03:EDZDA, 05:FBJCU; ‐DPA1*01:03:01, 02:01:01; ‐DPB1*04:EDJXU, 17:AHPBH. The most similar previously described allele is HLA‐A*02:01:01:01. Comparative sequence analysis revealed a single nucleotide substitution at position 1025 (codon 318; GGG → GAG), resulting in an amino‐acid substitution from Glycine to Glutamic Acid (G → E) (Figure 1A,B). The sequence has been submitted to GenBank under accession number PV434174.

**FIGURE 1:**
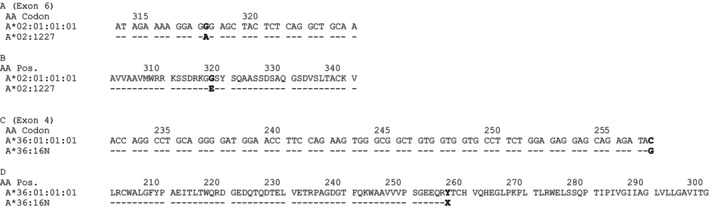
Sequence alignments illustrating the molecular differences between the novel and reference HLA‐A alleles. (A) Exon 6 nucleotide sequence alignment of HLA‐A*02:01:01:01 and the novel allele HLA‐A*02:1227. (B) Corresponding amino acid sequence alignment of HLA‐A*02:01:01:01 and HLA‐A*02:1227. (C) Partial exon 4 nucleotide sequence alignment of HLA‐A*36:01:01:01 and the novel null allele HLA‐A*36:16N. (D) Corresponding amino acid sequence alignment of HLA‐A*36:01:01:01 and HLA‐A*36:16N, showing the introduction of a premature stop codon. Nucleotide and amino acid positions are indicated above the sequences, and dashes denote identity with the A*02:01:01:01 and A*36:01:01:01 alleles, respectively.

The second allele, HLA‐A36:16N, was identified in an individual with the following HLA typing: HLA‐A*24:02:01, 36:16N; ‐B*15:03:01, 35:01:01; ‐C*02:10:01, 04:01:01; ‐DRB1*13:02:01, 15:ATRXY; ‐DRB3*03:BHJV; ‐DRB5*01:EZRWB; ‐DQA1*01:02:01, 01:02:01; ‐DQB1*06:EZWJB, 06:09; ‐DPA1*02:01:01, 02:01:08; HLA‐DPB1*01:EZSWS, ‐13:CXKCR. The most closely related previously described allele is HLA‐A*36:01:01. A nucleotide substitution was detected at position 843 (codon 257), consisting of a TAC → TAG change that introduces a premature stop codon (Tyrosine to stop) (Figure 1C,D). This mutation characterises the allele as a Null allele. The sequence has been deposited in GenBank under accession number PV799302. Ethnicity information was not available for either individual.

The names HLA‐A*36:16N and HLA‐A*02:1227 were officially assigned by the WHO Nomenclature Committee for Factors of the HLA System in May 2025 and August 2025, respectively. This follows the agreed policy that, subject to the conditions stated in the most recent Nomenclature Report, names will be assigned to new sequences as they are identified. Lists of such new names will be published in the following WHO Nomenclature Report [6].

## Author Contributions

**Augusto Cesar Soares dos Santos Júnior:** data analysis, manuscript writing and manuscript review. **Cíntia Keila Fabreti de Oliveira:** technical work, data analysis and manuscript review. **Raquel Aparecida Fabreti‐Oliveira:** sequence submission and manuscript review. All authors have read and approved the final manuscript.

## Funding

The authors have nothing to report.

## Conflicts of Interest

The authors declare no conflicts of interest.

## Data Availability

The data that support the findings of this study are openly available in IPD‐IMGT/HLA Database at https://www.ebi.ac.uk/ipd/imgt/hla/.
